# Retraction: Ophthalmic Combination of SurR9-C84A and Trichostatin-A Targeting Molecular Pathogenesis of Alkali Burn

**DOI:** 10.3389/fphar.2017.00426

**Published:** 2017-06-21

**Authors:** Frontiers Editorial Office

The Journal and Chief Editors retract the 28 July 2016 article cited above. Based on information discovered after publication and reported to Frontiers in November 2016, the article was examined, revealing image duplication in Figures 4A,B.

As this duplication breaches Frontiers guidelines and could not be sufficiently explained by the authors, the article has been retracted. The Editors acknowledge the authors' statement that these errors were the result of human error. The retraction of the article was approved by the Field Chief Editor for Frontiers in Pharmacology. The authors agree to the retraction and the notice.

